# Interferon beta 1b following natalizumab discontinuation: one year, randomized, prospective, pilot trial

**DOI:** 10.1186/1471-2377-13-101

**Published:** 2013-08-02

**Authors:** Claudio Gobbi, Dominik S Meier, François Cotton, Martina Sintzel, David Leppert, Charles R G Guttmann, Chiara Zecca

**Affiliations:** 1 Neurocenter of Southern Switzerland, Ospedale Regionale di Lugano, Lugano, Switzerland; 2 Center for Neurological Imaging, Departments of Radiology and Neurology, Brigham & Women’s Hospital, Harvard Medical School, Boston, MA, U.S.A; 3 Laboratoire d’Anatomie de Rockefeller, Lyon et Hospices civils de Lyon, Centre Hospitalier Lyon Sud, Service de Radiologie, Université de Lyon, Université Lyon 1, 69495, Pierre Bénite Cedex, France; 4 CREATIS-LRMN, CNRS UMR 5220-INSERM U630, Université de Lyon, Université Lyon 1, 69621, Villeurbanne Cedex, France; 5 MCS Medical Communication Services, Küsnacht, Switzerland; 6 Neurology, Department of Medicine, University Hospital Basel, Basel, Switzerland

**Keywords:** Multiple sclerosis, Natalizumab, Interferon beta1b, De-escalation, Progressive multifocal leukoencephalopathy

## Abstract

**Background:**

Natalizumab (NTZ) discontinuation leads to multiple sclerosis reactivation.

The objective of this study is to compare disease activity in MS patients who continued on NTZ treatment to those who were switched to subcutaneous interferon 1b (IFNB) treatment.

**Methods:**

1-year randomized, rater-blinded, parallel-group, pilot study (ClinicalTrial.gov ID: NCT01144052). Relapsing remitting MS patients on NTZ for ≥12 months who had been free of disease activity on this therapy (no relapses and disability progression for ≥6 months, no gadolinium-enhancing lesions on baseline MRI) were randomized to NTZ or IFNB. Primary endpoint was time to first on-study relapse. Additional clinical, MRI and safety parameters were assessed. Analysis was based on intention to treat.

**Results:**

19 patients (NTZ n=10; IFNB n=9) with similar baseline characteristics were included. 78% of IFNB treated patients remained relapse free (NTZ group: 100%), and 25% remained free of new T2 lesions (NTZ group: 62.5%). While time to first on-study relapse was not significantly different between groups (p=0.125), many secondary clinical and radiological endpoints (number of relapses, proportion of relapse free patients, number of new T2 lesions) showed a trend, or were significant (new T2 lesions at month 6) in favoring NTZ.

**Conclusions:**

De-escalation therapy from NTZ to IFNB over 1 year was associated with some clinical and radiological disease recurrence. Overall no major safety concerns were observed.

## Background

Natalizumab (NTZ) is an effective treatment for relapsing-remitting multiple sclerosis (RRMS), but is associated with an increased risk of progressive multifocal leucoencephalopathy (PML) in JC virus (JCV) sero-positive patients in function of treatment duration and pre-exposure to immunosuppressants [1]. Based on current risk benefit assessment [1], many physicians consider stopping treatment after 1–2 years. Mean half-life of unbound NTZ after repeated administrations is 11 days, and the drug is fully cleaned from the circulation within approximately 2 months after last infusion [2]. NTZ cessation may be followed by recurrence of disease activity peaking 4–7 months later in a significant number of patients, predominantly at MRI level [3,4]. Data providing guidance on the management of these patients, who generally suffer from rapidly evolving MS, are scarce. It had been suggested that if alternate treatment could minimize the risk of clinical flares, then NTZ dosage interruption might be an option for PML prevention [5].

The objective of this pilot study was to generate initial prospective data and hypotheses on the concept of de-escalating NTZ-treated patients with RRMS to interferon beta-1b (IFNB).

## Methods

This is a 1-year, prospective, controlled, randomized, rater blinded, parallel-group, monocentric pilot study (ClinicalTrial.gov ID: NCT01144052). Included patients were females or males with RR-MS according to 2005 McDonald’s criteria, aged between 18 and 60 years, who were on NTZ and feared or were at significant risk for PML. Risk for PML was defined significant in case of NTZ treatment duration equal to or greater than 12 months. Patients had to be free of disease activity while on NTZ (free from relapses and disability progression for at least 6 months and no gadolinium enhancing lesions [Gd+L] on baseline [BL] MRI). Main exclusion criteria were pregnancy and breastfeeding; relevant neurologic, internistic or psychiatric disorders; treatment with steroids less than 1 month before study entry; treatment with any immunomodulators or immunosuppressors other than steroids, ACTH or NTZ in the past year; inability to provide consent or comply with study procedures, current participation to other clinical trials. All the patients fulfilling inclusion and exclusion criteria and treated at our center were contacted and offered to participate.

Included patients were randomly assigned in a 1:1 ratio to continue monthly intravenous NTZ 300 mg or to de-escalate to every other day subcutaneous (s.c.) INFB 250 ug. INFB was started within 30 days after the last NTZ infusion. Patients intolerant to INFB were allowed to switch to daily s.c. glatiramer acetate (GA) 20 mg (rescue therapy).

Primary endpoint was time to first on-study relapse from randomization. Secondary endpoints included number of relapses, proportion of relapse free patients, severity of relapses (severe relapse was defined by ≥1.5 increase in EDSS score), 3 months confirmed disability progression (defined by ≥1.0 increase in EDSS score), number of new T2-hyperintense lesions (nT2L) and Gd+L per patient at months 3, 6, 9 and 12.

EDSS and relapses assessment was performed by an examining neurologist blinded to treatment. A relapse was defined according to widely accepted international diagnostic and therapeutic guidelines [6] as newly developing neurological symptoms or reactivation of pre-existing neurological deficits for a minimum of 24 hours in the absence of an increase in body temperature or infections occurring at least 30 days after the preceding episode. Relapses were confirmed when an increase of at least 1 point in at least one functional system was recorded. The occurrence of fatigue, mental symptoms, and/or vegetative symptoms without any additional signs was not classified as a relapse.

MRI disease activity was assessed via subtraction MRI (sMRI) [7] and via count of Gd+L by an expert who was blinded to clinical data.

Safety monitoring included physical examination, registration of adverse events, laboratory analysis and quarterly brain MRI.

Patients were consecutively recruited at the Neurocenter of Southern Switzerland, from 2010 to 2011. A monitoring agency prepared the randomization list and provided sealed envelopes for treatment allocation.

The study protocol is in compliance with the Helsinki Declaration and was approved by the local ethics committee and Swissmedic. Patients provided written informed consent before study enrollment. Written informed consent was obtained from the patient for the publication of this report and any accompanying images.

Statistical analyses were performed using non-parametric tests for continuous variables and ordinal scores, and considered significant at the level α=0.05. Analysis was based on intention to treat. This pilot study was conducted to generate first data and hypotheses for the planning of further clinical trials. The sample size was set to 20 patients, i.e. 10 patients per group and was based on clinical and practical considerations.

## Results

### Clinical findings

A total of 39 patients were screened, 25 fulfilled the inclusion and exclusion criteria and were offered to participate. Six patients refused their consent, 19 patients were included (NTZ n=10; IFNB n=9). No significant differences between treatment arms for baseline characteristics, including annualized relapse rate (ARR) during the 2 years prior to the NTZ run-in period, duration of run-in period of NTZ treatment and EDSS at randomization, were found (Table 1).

**Table 1:** Baseline characteristics of patients

	**IFNB (n=9)**	**NTZ (n=10)**	**p-value**
Females, n (%)*	3 (33%)	6 (60%)	0.370
Age, [years] ‡	39 (24–48)	43 (20–60)	0.460
Disease duration, [years] ‡	12 (2–23)	10 (5–17)	0.712
Number of NTZ infusions (run-in period) at baseline‡	21 (12–49)	25.5 (13–45)	0.661
Annualized relapse rate ‡			
- during 2 years prior to run-in period NTZ	1 (0.5-2.5)	1.3 (0.5-2.5)	0.661
- during run-in period NTZ	0 (0)	0(0–1.3)	0.497
EDSS ‡			
- during 2 years prior to run-in period NTZ	2 (1–3.5)	2.5 (1–3.5)	0.616
- at randomization	3 (1.5-3.5)	3 (1.5-3.5)	0.714
Therapy before run-in therapy with NTZ n (%) *			
- no treatment	1 (11%)	2 (20%)	1.000
- Glatiramer acetate	1 (11%)	2 (20%)	1.000
- IFNbeta 1a im	1 (11%)	2 (20%)	1.000
- IFNbeta 1a sc	2 (22%)	3 (30%)	1.000
- IFNbeta 1b	4 (44%)	1 (10%)	0.141

*two-sided exact Fisher test, ‡ U-Mann–Whitney test; values are median (range).

17/19 patients completed the study: one IFNB-patient withdrew consent (day 34) because she could not comply with study procedures; one NTZ-patient opted for an oral treatment (day 139). One IFNB-patient (#9) switched to rescue treatment at day 69 due to systemic side effects.

Median time to first on-study relapse was 103 days in the IFNB group; no relapses were observed in the NTZ group (p=0.125) (Table 2). Seven out of 9 (78%) IFNB-patients remained relapse free, compared to all 10 in the NTZ group (p=0.206) over the 1-year follow-up. Two IFNB-patients experienced a total of 3 relapses (p=0.447), all with transitory EDSS worsening [0.5 points in patient #5 and #14 (both at month 4), 1.5 points in patient #14 (month 11)]. Both patients scored negative for neutralizing antibodies against IFNB. No patient experienced sustained disability progression. The ARR on study in both treatment groups was significantly lower compared to the period prior the run-in NTZ treatment (IFNB: p=0.034; NTZ: p=0.005).

**Table 2:** Study outcomes

	**IFNB (n=9)**	**NTZ (n=10)**	**p-value**
Median time to first on study relapse (primary endpoint)*	103 days	-	0.125
Number of relapses^	3	0	0.447
Proportion of relapse free patients (number)°	78% (7)	100% (10)	0.206
Severity of relapses:			
- EDSS score change, median (range)	0.5 (0.5 -1.5)	-	-
Number of patients with 3 months confirmed disability progression°:			
*(1 patient showed a disability progression of 1.5 points 1 month after an attack occurred during month 11 of study)*	0	0	1
Number of nT2L, median (range) ^			
- at month 3	0.5 (0–2)	0 (0–1)	0.146
- at month 6	1.5 (0–9)	0 (0–2)	0.043
- at month 9	0.5 (0–6)	0 (0)	0.105
- at month 12	0 (0–12)	0 (0)	0.234
Number of Gd+L, median (range) ^			
- at month 3	0 (0–1)	0 (0)	0.696
- at month 6	0 (0–5)	0 (0)	0.442
- at month 9	0 (0–1)	0 (0)	0.694
- at month 12	0 (0–2)	0 (0–1)	0.694
Adverse events			
- number of infection, median (range) ^	0	1	0.140
- proportion of patients with at least 1 infection °	3 (33%)	7 (70%)	0.179
- number of patients with injection site reactions	4	n.a.	-

* Log-rank test ; ^U-Mann Whitney test; ° two-sided exact Fisher test.

Injection site reactions occurred in 44% of IFNB-patients. The median number of infections per patient was not statistically different between groups (IFNB = 0, NTZ = 1, p=0.140) (Table 2). There were no case of immune reconstitution inflammatory syndrome.

### MRI findings

**Figure 1:**
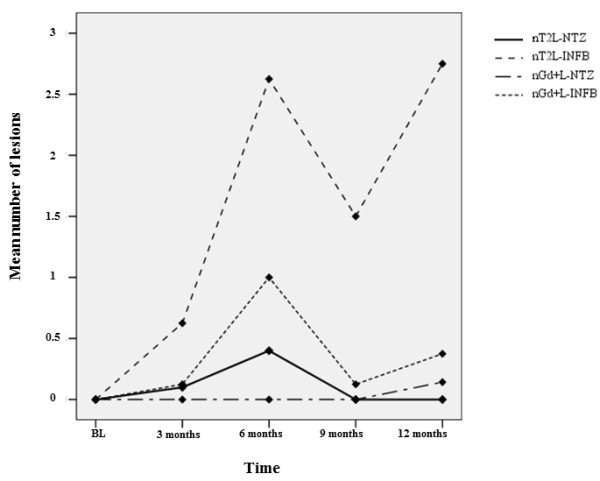
**Mean number of new T2 lesions (nT2L) and gadolinium enhancing lesions (Gd+L) per patient at baseline and at month 3, 6, 9 and 12 of study (statistical analysis was performed with non parametric tests and reported in Results and Table **2**).**

**Figure 2:**
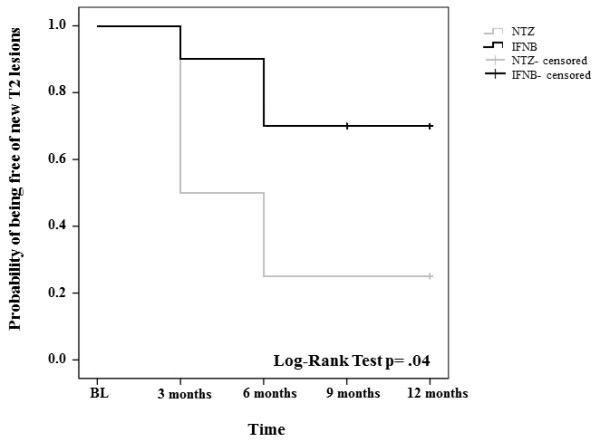
**Kaplan Meier survival curves representing the probability of being free of nT2L lesions throughout the study period under de**-**escalation therapy with INFB or continued NTZ.**

A higher number of nT2L was detected in the IFNB vs. the NTZ group at each time point (Figure 1), this difference being significant only for time point month 6 (Table 2). However, the overall likelihood to remain free of nT2L was significantly higher with NTZ (62.5%) than with IFNB therapy (25%) (Figure 2). The number of Gd+L per patient did not significantly differ between groups at any time point (Table 2).

No associations were found between ARR, nT2L and Gd+L during the study period and demographic or clinical parameters shown in Table 1.

## Discussion and conclusions

We reported the effects on clinical disease activity and MRI surrogates in MS patients treated with NTZ who either switched to IFNB or continued on NTZ in a prospective, one-year, randomized, rater-blinded study. The main result of our study is that clinical and radiological disease activity was larger in the de-escalating group, and that only 25% of patients treated with IFNB were free from disease recurrence (relapses, disability progression and nT2 brain lesions) after one year. The primary endpoint (time to first on study relapse) showed a clear trend in favor of continued NTZ treatment, however, statistical significance was not reached probably due to the small sample size. Moreover, given that IFNB was started within 30 days of cessation of NTZ, there may have been residual efficacy of NTZ for up to 3 months in the IFNB group [8]. Also, relapses were not associated with neutralizing antibodies against IFNB, which have been associated with IFNB treatment failure.

Clinical results are supported by MRI findings showing a statistically significant greater number of nT2L at month 6 and a coherent trend in favor of NTZ treatment for all the remaining radiological endpoints. The higher number of nT2L in the IFNB vs. the NTZ group at each time point, and higher number of patients free of nT2L in the NTZ treated arm indicate a higher efficacy of NTZ. This is in line with previous reports, showing that radiological reactivation peaks at months 4–7 following NTZ discontinuation [3].

INFB, although not sufficient to protect from disease recurrence, seems to exert a certain anti-inflammatory activity after NTZ discontinuation: the three relapses occurring in the IFNB group were mild and did not result in sustained progression over 6 months. Also, there were no cases of dramatic clinical worsening referable to immune reconstitution inflammatory syndrome sometimes described after NTZ discontinuation [3,4,9-11]. Moreover, as many as 77% (7/9) patients were free from recurrence of clinical activity, and 25% from recurrence of radiological activity over one year; the majority of patients had only one or two nT2L, and only three patients showed three or more nT2 lesions.

The only randomized trial on NTZ de-escalation including interferon beta as a comparator is the RESTORE study [12], which evaluated the effect of a 24 week interruption in NTZ treatment comparing continued NTZ treatment with placebo or with switching to GA, i.m interferon beta 1a or methylprednisolone. The preliminary data of the trial showed a high rate of recurrence of MRI and clinical disease activity following NTZ discontinuation. However, this study was limited by partial randomization and imbalanced baseline characteristics of patients, and was not powered to detect significant differences among treatment groups. Our results are in line with the reduced early reactivation risk following discontinuation of NTZ observed with early GA treatment, which was recently described by Rossi et al. [13] in a single-arm study limited by the absence of a control arm. On the other hand, in two small observational studies GA following NTZ cessation was followed by severe recurrence of disease activity [9,14]. Possible explanations of these apparently contrasting results may reflect the delayed onset of action of GA [15] particularly considering that studies employed different wash out intervals before switching from NTZ to GA.

Of note, the ARR under de-escalation therapy with IFNB was significantly lower than in the two years prior to the run-in NTZ therapy. While this may reflect the natural disease course, statistical regression to the mean, differences among first line DMTs used before NTZ, or an induction effect by NTZ [3,5], it might also suggest that IFNB could represent an alternative treatment for a selected subgroup of patients de-escalating NTZ treatment. In an attempt to define factors that would allow predicting IFNB responders, we analyzed associations between on-study disease activity with demographic, clinical and radiological parameters prior to run-in NTZ therapy (data not shown). However, we could not identify such biomarkers yet, probably reflecting the small sample.

Our study has limitations. Firstly, the small sample size; nonetheless, the study has a frequent MRI monitoring to catch subclinical disease activity and increase sensitivity for disease recurrence. Moreover, our sample is fully representative of the MS population treated with NTZ in southern Switzerland as all patients receiving NTZ in this region are treated at our Center. Clinical and radiological characteristics of patients treated with NTZ at our Center who were included or not in the study were similar (data not shown). Sixty-three percent of our patients were free from any radiological activity (i.e. Gd+L and nT2L lesions) under NTZ treatment, analogous to the data reported for the first year of the AFFIRM study [16]. Moreover, our patients experienced over 90% reduction of ARR under NTZ treatment compared to the two years before NTZ initiation, which is in line with the known efficacy of NTZ in active MS patients [3]. Taken together, these data suggests that our study population is similar to NTZ treated populations reported in the literature. The absence of a placebo arm represents the second limitation of the study but was expressly avoided for ethical considerations. Thirdly, patients were not stratified according to anti JC virus antibodies testing, which was not validated at the time when our study was conducted.

Besides the frequent MRI monitoring and the highly sensitive MRI metrics, the main strength of our study is the prospective, randomized design. The study length can be considered as appropriate given that NTZ fully clears from the circulation in approximately two months (5 half-lives) and CSF lymphocyte count remains suppressed up to 6 months following the last dose [16]. Finally, our study was conducted without third-party funding.

A larger trial is warranted to confirm present results and possibly identify predictive markers to define that segment of patients who is likely to be protected best with de-escalation therapy with IFNB. This would mark a significant advantage for a patient population that otherwise runs a potentially increasing risk for PML with continued NTZ therapy.

## Competing interests

Claudio Gobbi has received personal compensation from Teva, Merck Serono, Biogen Idec, Bayer Schering, Novartis. D. Meier has nothing to disclose. Francois. Cotton has nothing to disclose. Martina Sintzel or her agency has received consulting fees and/or honoraria from Bayer Health Care Pharmaceuticals, Fresenius Medical Care, UCB, Ospedale Regionale di Lugano and ETH Zurich. Charles R.G. Gutmann has received personal compensation from Tibotec/Johnson & Johnson and was the recipient of a research grant from Teva Neuroscience, within the last 3 years. David Leppert is an employee of F. Hoffmann – La Roche Ldt. Chiara Zecca has received personal compensation from Teva, Merck Serono, Biogen Idec, Bayer Schering, Novartis.

## Authors’ contributions

CG has contributed to the design/conceptualization of the study, analysis/interpretation of the data, and drafting/revising the manuscript for intellectual content. DM has contributed to the design/conceptualization of the MRI component of the study, image review, result collection and the analysis/interpretation of the data, and revising of the manuscript for intellectual content. FC has contributed to the analysis/interpretation of the MRI data. MS has contributed to the design/conceptualization of the study and revising the manuscript for intellectual content. DL has contributed to the analysis/interpretation of the data, and revising the manuscript for intellectual content. CRGG has contributed to the design/conceptualization of the MRI component of the study and the analysis/interpretation of the data, and revising of the manuscript for intellectual content. CZ has contributed to the design/conceptualization of the study, analysis/interpretation of the data, and drafting/revising the manuscript for intellectual content. All authors read and approved the final manuscript.

## Pre-publication history

The pre-publication history for this paper can be accessed here:

http://www.biomedcentral.com/1471-2377/13/101/prepub
